# Predictive performance and verification of physiologically based pharmacokinetic model of propylthiouracil

**DOI:** 10.3389/fphar.2022.1013432

**Published:** 2022-10-05

**Authors:** Chaozhuang Shen, Dahu Liang, Xiaohu Wang, Wenxin Shao, Kuo Geng, Xingwen Wang, Hua Sun, Haitang Xie

**Affiliations:** ^1^ Graduate School, Wannan Medical College, Wuhu, Anhui, China; ^2^ Anhui Provincial Center for Drug Clinical Evaluation, Yijishan Hospital of Wannan Medical College, Wuhu, Anhui, China

**Keywords:** propylthiouracil (PTU), PBPK, PK-sim^®^, extrapolation, elderly, pediatric

## Abstract

**Background:** Propylthiouracil (PTU) treats hyperthyroidism and thyroid crisis in all age groups. A variety of serious adverse effects can occur during clinical use and require attention to its pharmacokinetic and pharmacodynamic characteristics in various populations.

**Objective:** To provide information for individualized dosing and clinical evaluation of PTU in the clinical setting by developing a physiologically based pharmacokinetic (PBPK) model, predicting ADME characteristics, and extrapolating to elderly and pediatric populations.

**Methods:** Relevant databases and literature were retrieved to collect PTU’s pharmacochemical properties and ADME parameters, etc. A PBPK model for adults was developed using PK-Sim® software to predict tissue distribution and extrapolated to elderly and pediatric populations. The mean fold error (MFE) method was used to compare the differences between predicted and observed values to assess the accuracy of the PBPK model. The model was validated using PTU pharmacokinetic data in healthy adult populations.

**Result:** The MFE ratios of predicted to observed values of AUC_0-t_, C_max_, and T_max_ were mainly within 0.5 and 2. PTU concentrations in various tissues are lower than venous plasma concentrations. Compared to healthy adults, the pediatric population requires quantitative adjustment to the appropriate dose to achieve the same plasma exposure levels, while the elderly do not require dose adjustments.

**Conclusion:** The PBPK model of PTU was successfully developed, externally validated, and applied to tissue distribution prediction and special population extrapolation, which provides a reference for clinical individualized drug administration and evaluation.

## 1 Introduction

Propylthiouracil (PTU) is a thiourea antithyroid drug that inhibits the synthesis of thyroid hormones by inhibiting the peroxidase system in the thyroid gland and preventing the iodination of tyrosine and condensation of iodinated tyrosine in the thyroid gland. It is used clinically to treat hyperthyroidism and thyroid crisis (Amisha and Rehman, 2022). In clinical practice, PTU is used in all age groups. The concern is that PTU can cause a variety of adverse effects, including hepato-toxicity (Gomez-Peralta et al., 2018), granulocyte deficiency (Tomkins et al., 2020), vasculitis (Hiruma et al., 2021), and hypothyroidism (Piantanida et al., 2020). Cases of liver failure, liver transplantation, and even death due to liver injury have been reported in adult and pediatric patients treated with PTU (Williams et al., 1997). It has been reported that the plasma concentration of PTU correlates with clinical efficacy and adverse effects, thus, drug monitoring should be conducted around the whole dosing cycle of PTU (Amisha and Rehman, 2022).

The distribution and accumulation of drugs in the body may lead to abnormal tissue function, which is one of the crucial reasons for various serious adverse reactions of many drugs. The tissue distribution of drugs in the human body is difficult to obtain through clinical trials, and previous pharmacokinetic studies mainly focused on the exposure of drugs in blood. Therefore, we used a modeling approach to predict drug concentrations in various tissues to provide valuable reference for further studies.

The rational use of drugs in special populations such as pediatric, elderly, pregnant and hepatic and renal insufficiency is an urgent problem for clinical pharmacologists and clinicians worldwide. Due to the significant individual differences in pediatric and elderly populations, ethical and practical constraints have led to limitations in conducting clinical trials when evaluating the efficacy and safety of medications. Approximately 60%–90% of children have used drugs that are not explicitly mentioned in the instructions for use in the pediatric population, and this percentage is even higher than 90% in infants and children under 1 year of age (Hsien et al., 2008; Leong et al., 2012). The elderly often has a variety of diseases which lead to organ hypofunction, especially in the liver and kidney, the two major organs responsible for drug metabolism and elimination (Stader et al., 2019).

At present, traditional pharmacokinetic studies and modeling technology are more and more closely combined. The physiologically based pharmacokinetic (PBPK) model is defined as a mathematical model that simulates the concentration of a drug over time in tissues and blood by considering the rate of the drug’s absorption, distribution, metabolism, and excretion (ADME) based on the interplay between physiological, physicochemical and biochemical determinants (Pang and Durk, 2010; Grimstein et al., 2019). It allows for PK estimation with limited data by using existing physiological/physicochemical information and is mainly used to enable *in vitro* to *in vivo* prediction, simulation of tissue distribution characteristics of drugs *in vivo*, interspecies scaling and extrapolation for special populations, etc. Moreover, the US Food and Drug Administration (FDA), the European Medicines Agency (EMA), and The International Council for Harmonisation of Technical Requirements for Pharmaceuticals for Human Use (ICH) have recommended the use of models to guide drug development and dose selection (EMA, 2018; FDA, 2020; ICH, 2022).

**FIGURE 1:**
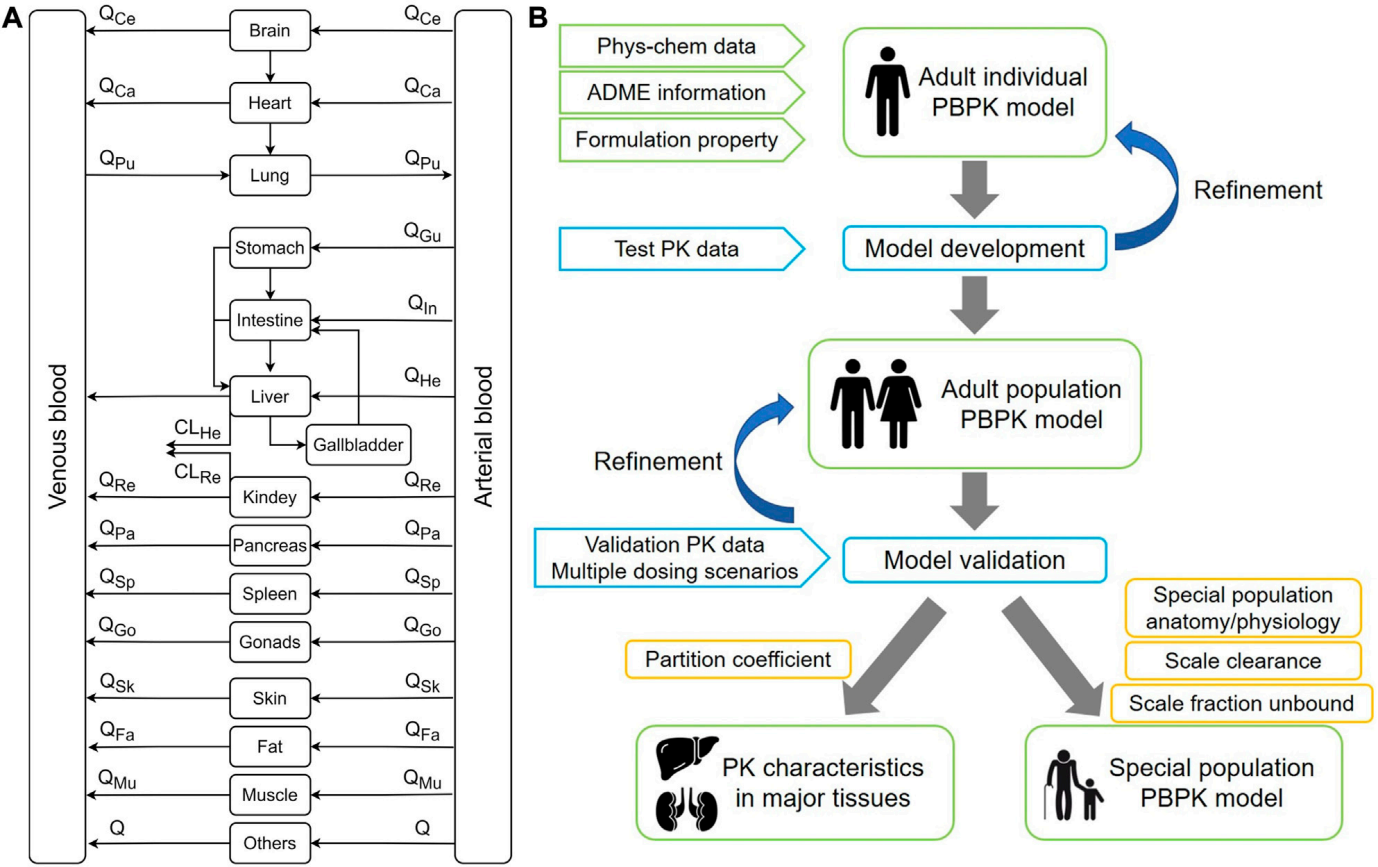
The overall design of this modeling study. **(A)** A whole-body physiologically-based pharmacokinetic model. Q refers to blood flow. **(B)** Diagram for modeling workflow.

In recent years, PBPK models have been developed in various fields of drug applications and have been widely applied to the prediction of drugs in special populations, including pediatrics, pregnancy, and the elderly (Templeton et al., 2018; Cui et al., 2021; Szeto et al., 2021). PBPK modeling method is utilized to evaluate drug safety and efficacy by enriching drug information in special populations, guiding the clinical drug usage and reducing unnecessary clinical trials (Lin et al., 2022). Therefore, this study makes full use of data acquired from existing clinical studies of PTU to develop and validate the PBPK model, predict the tissue distribution characteristics, and extrapolate to pediatric and elderly populations to provide a reference for individualized dosing and clinical evaluation. An overview of the PBPK model structure and general modeling workflow is depicted in Figure 1.

## 2 Materials and methods

### 2.1 Modeling parameter collection and processing

PBPK model development used Open Systems Pharmacology Suite incorporating PK-Sim® (Version 11, Open Systems Pharmacology Suite, https://www.open-systems-pharmacology.org/), using the Monte Carlo algorithm for parameter identification. The physicochemical parameters of PTU were predicted and queried using ADMETlab (https://admet.scbdd.com/), SwissADME (http://www.swissadme.ch/), ADMETlab 2.0 (https://admetmesh.scbdd.com/), ADMESAR (http://lmmd.ecust.edu.cn/admetsar2/) and Drugbank (https://go.drugbank.com/) databases. Plasma concentration-time curve data from the literature were obtained for model validation using GetData Graph Digitizer (Version 2.25.0.32, S. Fedorov, http://www.getdata-graph-digitizer.com/) software. Pharmacokinetic parameters were calculated using the Phoenix WinNonlin 8.1 (Pharsight Corp. Mountain View, CA, United States, https://www.certara.com/software/phoenix-winnonlin/) non-compartmental model. Graph editing and processing were performed using GraphPad Prism 8.

### 2.2 Human bioequivalence trial design and result evaluation of PTU

The clinical data used to develop a PBPK model of 50 mg PTU tablets were obtained from the bioequivalence (BE) trial in healthy Chinese volunteers conducted by the Anhui Provincial Center for Drug Clinical Evaluation, Yijishan Hospital of Wannan Medical College. The study was approved by the Drug, Device, and New Technology Ethics Committee of Yijishan Hospital of Wannan Medical College. All subjects signed the informed consent form. The trial was performed abiding by the Declaration of Helsinki, Good Clinical Practice, and the guidelines of China National Medical Products Administration. The registration numbers of this study are B201800078-01 (www.chinadrugtrials.org.cn; announcement date, Feb. 25, 2018) and CTR20180444 (www.chinadrugtrials.org.cn; announcement date, 21 May 2018).

This was a randomized, open-label, two periods, two sequences, crossover, single-dose, fasting design trial. The 24 subjects received one propylthiouracil tablet test (T) formulation or reference (R) formulation (50 mg/tablet, Propylthiouracil Tablets®, DAVA Pharmaceuticals, Inc.) orally in each period. Subjects were randomly assigned to two groups, subjects in the first group administered in the order of T and R and subjects in the second group administered in the order of R and T. The washout period between each study period was 7 days. Plasma collection points were before (within 1 h) and 15 min (0.25 h), 30 min (0.5 h), 40 min (0.67 h), 50 min (0.83 h), 1.0 h, 1.25 h, 1.5 h, 1.75 h, 2 h, 2.33 h, 2.67 h, 3 h, 4 h, 5 h, 6 h, 8 h, and 12 h after PTU administration. All plasma samples were detected and analyzed using HPLC-MS/MS method (Mendes et al., 2014).

The pharmacokinetic data were analyzed using Phoenix WinNonlin software. The two one-sided *t* test was performed for C_max_, AUC_0-t_, and AUC_0-∞_ in 24 subjects after natural logarithmic conversion, and 90% confidence interval (CI) were calculated for the geometric mean ratios. When the 90% CI of the geometric mean ratios of C_max_, AUC_0-t_, and AUC_0-∞_ for the subject formulation and the reference formulation were within the 80.00%–125.00% equivalence interval, it was determined that the two formulations were bioequivalent, i.e., both formulations could be used for PTU adult 50 mg PBPK model development.

### 2.3 Adult PBPK model development and evaluation

An adult PBPK model of 50 mg PTU administered orally was developed using PK-Sim® software containing 18 compartment models. The organs were consisted of four sub-compartments (blood, plasma, interstitial, and intracellular) (Willmann et al., 2012). The PBPK model was constructed using the physicochemical properties, *in vitro* characteristics, and *in vivo* ADME information of PTU, as shown in Table 1, combining the information of BE trial subjects. We created a virtual individual of East Asian ethnicity using average age, weight, and height of 25.7 years, 60.98 kg, and 167.60 cm from BE trial, respectively, to simulate the mean plasma concentration vs time after taking 50 mg PTU. Meanwhile, based on the established individual, a virtual Asian population containing 100 individuals (50% female) was built by the “Population” module of the software to simulate the pharmacokinetic behavior of PTU in the study (Tanaka, 1996). According to relevant databases, parameters such as lipophilicity (log P), fraction unbound (fu), molecular weight, pka, and solubility of PTU were integrated and optimized. To simulate the disposition of PTU, we searched literatures for pharmacokinetic information of PTU, obtained the approximate range of total plasma clearance and calculated the hepatic and renal clearance. It has been reported that about 60% of PTU is metabolized in the liver and 35% of the drug is excreted in the urine in intact and bound form (Ebrahimzadeh et al., 2011; Amisha and Rehman, 2022). After determining initial values by visual fitting and comparing the differences between predicted values and pharmacokinetic data from BE trials, the parameters were optimized using Monte Carlo simulations and adjusted according to the actual clearance ratio of the drug. The final calculations were performed using PK-Sim® to calculate hepatic and renal specific clearance to simulate the process. The following formulas from PK-Sim® calculated hepatic and renal specific clearance:

$$Renal\;specific\;clearance=\frac{CL_{\mathrm{pls}}\times BW\times Q_{\mathrm{kid}}\times \left(1-HCT\right)}{fu\times \left(Q_{\mathrm{kid}}\times \left(1-HCT\right)-CL_{\mathrm{pls}}\times BW\right)\times V_{\mathrm{kid}}}:1000000$$ (1)

$$Hepatic\;specific\;clearance=\frac{CL_{\mathrm{pls}}\times BW\times \left(Q_{\mathrm{liv}}+Q_{\mathrm{pve}}\right)}{fu\times \left(Q_{\mathrm{liv}}+Q_{\mathrm{pve}}-\frac{CL_{\mathrm{pls}}}{B/P}\times BW\right)\times V_{liv}\times f_{cell}}:1000000$$ (2)

**TABLE 1:** Parameters required for PBPK model establishment.

Parameter type	Value	Source
**Species information**		
**Individual**		
Species	Human	—
Race	East Asian	—
Gender	Male	—
Endothelal Surface areas	Organ Vasculrization	PK-Sim standard
Body surface area	Mosteller	PK-Sim standard
Age (years)	25.7	Project mean
Weight (kg)	60.98	Project mean
Height (cm)	167.60	Project mean
BMI	21.71	Project mean
**Population**		
Age (years)	18–60	Simulation setting
Number of individuals	100	Simulation setting
Proportion of females	50%	Simulation setting
**Drug information**		
**Physiochemical properties**		
log P	0.4 (Drugbank)	Optimized value: 0.43
	0.421 (ADMETlab)	PK-Sim optimization
	0.935 (ADMETlab 2.0)	
	1.38 (SwissADME)	
	1.57 (ADMESAR)	
fu (plasma)	18% (Drugbank)	Drugbank
	28.64% (ADMETlab 2.0)	
	33.306% (ADMETlab)	
Moleculr weight	170.23	Drugbank
pka (Strongest Acidic)	8.09	Drugbank
pka (Strongest Basic)	-2.9	Drugbank
Solublity (mg/L)	1,200 (Drugbank)	Drugbank
	1,531.278 (ADMETlab)	
	234 (SwissADME)	
**Absorption**		
Specific intestinal permeability (cm/min)	3.92x10^−6^	Thelen et al. (2011)
**Distribution**		
Specific organ permeability (cm/min)	3.18x10^−3^	Willmann et al. (2005)
**Metabolism and Elimination**		
Total clearance (L/h)	4.42–13.5	Pan et al. (2001); Tang. et al. (2002); Mendes et al. (2014); Główka et al. (2018)
		Optimized value: 9
		PK-Sim optimization
Total clearance (L/h/kg)	0.07–0.22	—
Total hepatic clearance (L/h/kg)	0.09	PK-Sim optimization
Hepatic specific clearance (1/min)	0.36	PK-Sim calculation
Renal clearances (L/h/kg)	0.05	PK-Sim optimization
Renal specific clearance (1/min)	0.70	PK-Sim calculation
**Dosing Information**		
Administration type	Oral	—
Dose (mg)	50	—
Dosing interval	Single	—
Formulations	Dissolved	PK-Sim standard

CL_pls_ (L/h/kg): Plasma clearance; BW (kg): Body weight; Q_kid_ (L/min): Blood flow rate (kidney); fu: Fraction unbound; HCT: Hematocrit; V_kid_ (L): Volume (kidney); Q_liv_ (L/min): Blood flow rate (liver); Q_pve_ (L/min): Blood flow rate (portal vein); B/P: Blood/Plasma concentration ratio; V_liv_ (L): Volume (liver); f_cell_: Fraction intracellular (liver). Q_kid_, HCT, V_kid_, Q_liv_, Q_pve_, B/P, V_liv_, f_cell_ used the software default values.

The observed values are obtained in the BE study, and the PK parameters were calculated using the non-compartmental model analysis (NCA) in Phoenix WinNonlin software. The initial model was developed based on 50 mg reference formulation data, and the test formulation data were used for comparative evaluation. The population predictions’ mean and 90% CI were obtained using software simulations. The visual inspection method was used to compare the predicted and observed values to evaluate the model’s prediction performance. The mean fold error (MFE) method was used to compare maximum concentration (C_max_), time to maximum concentration (T_max_), and area under the curve from 0 to the time of the last quantified concentration (AUC_0-t_) to assess the model fit. The model was well developed when all pharmacokinetic parameters met 0.5 < MFE <2. (MFE = Predicted/observed values).

### 2.4 Model verification

For validation of the model, it was refined and optimized using the clinical plasma concentration data obtained in healthy adults following oral administrations of PTU. The data were retrieved from two clinical BE trials using doses of 100 mg and 200 mg, respectively (Tang. et al., 2002; Mendes et al., 2014). We developed the PBPK model for oral PTU 100 mg and 200 mg without changing parameters other than the dose and compared AUC_0-t_, C_max_, and T_max_ of predicted and observed values.

### 2.5 Prediction of pharmacokinetic behavior in major tissues

To further understand the pharmacokinetic behavior characteristics of PTU in tissues, we simulated the plasma drug concentration-time profiles of 50 mg PTU in major organs to characterize the absorption and distribution of PTU *in vivo*. The tissue concentrations of major organs in the human body were simulated, including brain, heart, liver, spleen, lung, kidney, stomach, and muscle. Comparing the differences in drug concentrations in each tissue with venous plasma.

### 2.6 Elderly and pediatric virtual populations PBPK model extrapolation and evaluation

To simulate the pharmacokinetic behavior of 50 mg PTU in elderly and pediatric populations, we extrapolated the oral PBPK model in virtual elderly and pediatric populations based on validated adult PBPK models. The algorithms implemented in PK-Sim® were used to generate the virtual population (Willmann et al., 2007). The population was grouped according to the standard age groups, including term infants (0–27 days), infants (28 days–23 months), children (24 months–11 years), adolescents (12 years–17 years), and elderly (60 years–81 years) by taking into the account of the age-related physiological changes in elderly and children (NMPA, 2014). Each virtual group consisted of 50 females and 50 males.

During the creation of the virtual population and simulation of the PBPK model, physiological information related to age, including blood flow to different organs, GIT radius, length, and effective surface area, were scaled by PK-Sim® software according to the age groups (ICRP, 2002; Edginton et al., 2006; Schlender et al., 2016). Other scalable parameters, such as gastric emptying time, intestinal transit time, and gastric pH, were set at the default values (Kawai et al., 1994; Rodgers et al., 2005; Thelen et al., 2011). We mainly considered scaling of hepatic clearance, renal clearance and fu for extrapolation of drug-specific parameters. The allometric scaling model extrapolated the clearance in adults to the elderly and pediatric populations. Clearance increased nonlinearly with body weight at a rate of 0.75. The formula is as follows (Liang. et al., 2020):

$$CL_{tar}=CL_{ad}*{\left(\frac{W_{tar}}{W_{ad}}\right)}^{0.75}$$ (3)

CL_tar_ and W_tar_ represent clearance and body weight in the target population; CL_ad_ and W_ad_ represent clearance and body weight in adults.

*In vivo*, only free drugs can be transported across membranes and contribute to efficacy. The plasma protein binding rate is a crucial factor affecting the number of free drugs in the body. The body levels of albumin (Alb), a protein closely related to plasma protein binding, change with age. By determining the plasma protein concentration in the body, the percentage of free drugs in the body can be predicted, which is calculated as following formulas (Wallace, 1976; Kuhnz and Nau, 1983; Veering et al., 1990; McNamara and Alcorn, 2002):

Alb(g/L)=1.1287×ln(Age)+33.746 (4)

$${fu}_{tar}=\frac{1}{\left(1+\left(1-{fu}_{ad}\right)\times \frac{P_{tar}}{P_{ad}\times {fu}_{ad}}\right)}$$ (5)

fu_tar_ and P_tar_ (g/L) represent fu and plasma protein concentrations in the target population; fu_ad_ and P_ad_ (g/L) represent fu and plasma protein concentrations in adults. The demographic information of the population and the drug-specific parameters after extrapolation are shown in Table 2.

**TABLE 2:** Demographic information and drug-specific parameters in virtual elderly and pediatric populations.

Parameter type	Adult	Elderly	Term infant	Infant	Children	Adolescent
**Individual**						
Age (year)	25.7	70.5	13.5 days	1	6.5	14.5
Weight (kg)	60.98	58.04	4.28	10.66	21.46	51.37
Height (cm)	167.60	165.40	54.11	81.49	118.94	162.81
BMI (kg/m^2^)	21.71	21.21	14.63	16.05	15.17	19.38
fu (%)	18	17.56	21.47	19.57	18.63	18.26
**Population**						
Age (years)	18–60	60–81	0–27 days	28 days-23 months	1–11	12–17
Number of individuals	100	100	100	100	100	100
Proportion of females	50%	50%	50%	50%	50%	50%
**Metabolism and Elimination**						
Total Clearance (L/h)	9	8.672	1.23	2.433	4.112	7.914
Total clearance (L/h/kg)	0.147	0.149	0.287	0.228	0.192	0.154
Total Hepatic Clearance (L/h/kg)	0.09	0.0896	0.17	0.14	0.12	0.092
Hepatic Specific Clearance (1/min)	0.36	0.34	0.04	0.09	0.15	0.30
Renal Clearances (L/h/kg)	0.05	0.0523	0.10	0.08	0.07	0.054
Renal Specific Clearance (1/min)	0.70	0.69	0.09	0.07	0.31	0.63

## 3 Result

### 3.1 Bioequivalence evaluation

**FIGURE 2:**
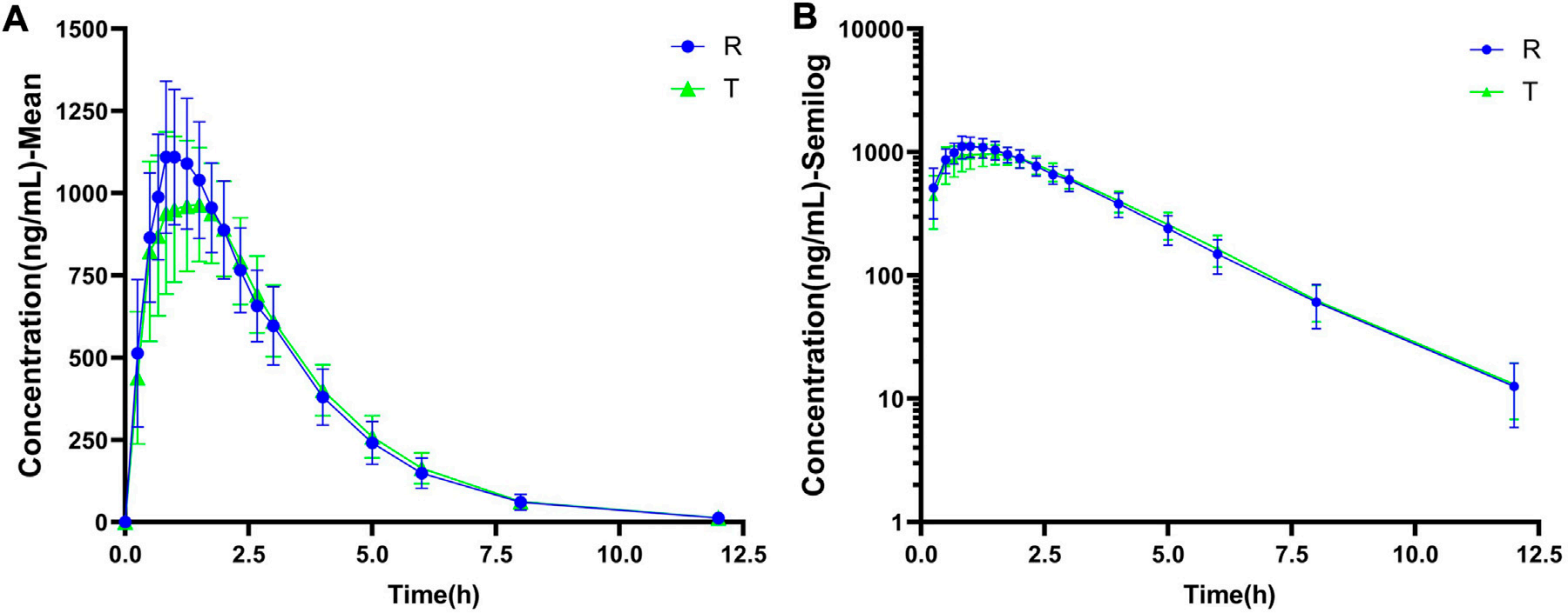
The observed data of plasma drug concentration-time curve of oral PTU. **(A)** Mean plasma concentration-time curve. **(B)** Semi-log curve. Blue circles: reference formulation (R). Green triangles: test formulation (T). Data are presented as mean ± standard deviation.

The linear and semi-logarithmic scales of the plasma concentration-time curves of PTU in groups T and R are shown in Figure 2. As shown, the mean plasma concentrations of PTU in groups T and R were close at all time points without significant differences. The results of the bioequivalence analysis showed that the 90% CI for the T/R ratios of C_max_, AUC_0-t_, and AUC_0-∞_ for both the T and R groups were within the 80.00%–125.00% equivalence interval (Table 3). The results satisfy the bioequivalence conditions and can be used to fit the adult PBPK model.

**TABLE 3:** Statistical results of bioequivalence (*n* = 24).

Parameter	Number	Geometric mean		Geometric mean ratio (%)	90%CI	Individual coefficient of variation (%)	Power (1-β, %)
		R	T				
AUC_0-∞_ (ng*h/mL)	24	3699.9	3627.1	98.03	(95.40–100.73)	5.49	100.0
AUC_0-t_ (ng* h/mL)	24	3667.3	3592.9	97.97	(95.32–100.69)	5.54	100.0
C_max_ (ng/ml)	24	1253.8	1129.4	90.08	(82.73–98.08)	17.29	75.0

### 3.2 PBPK modeling and model evaluation

**FIGURE 3:**
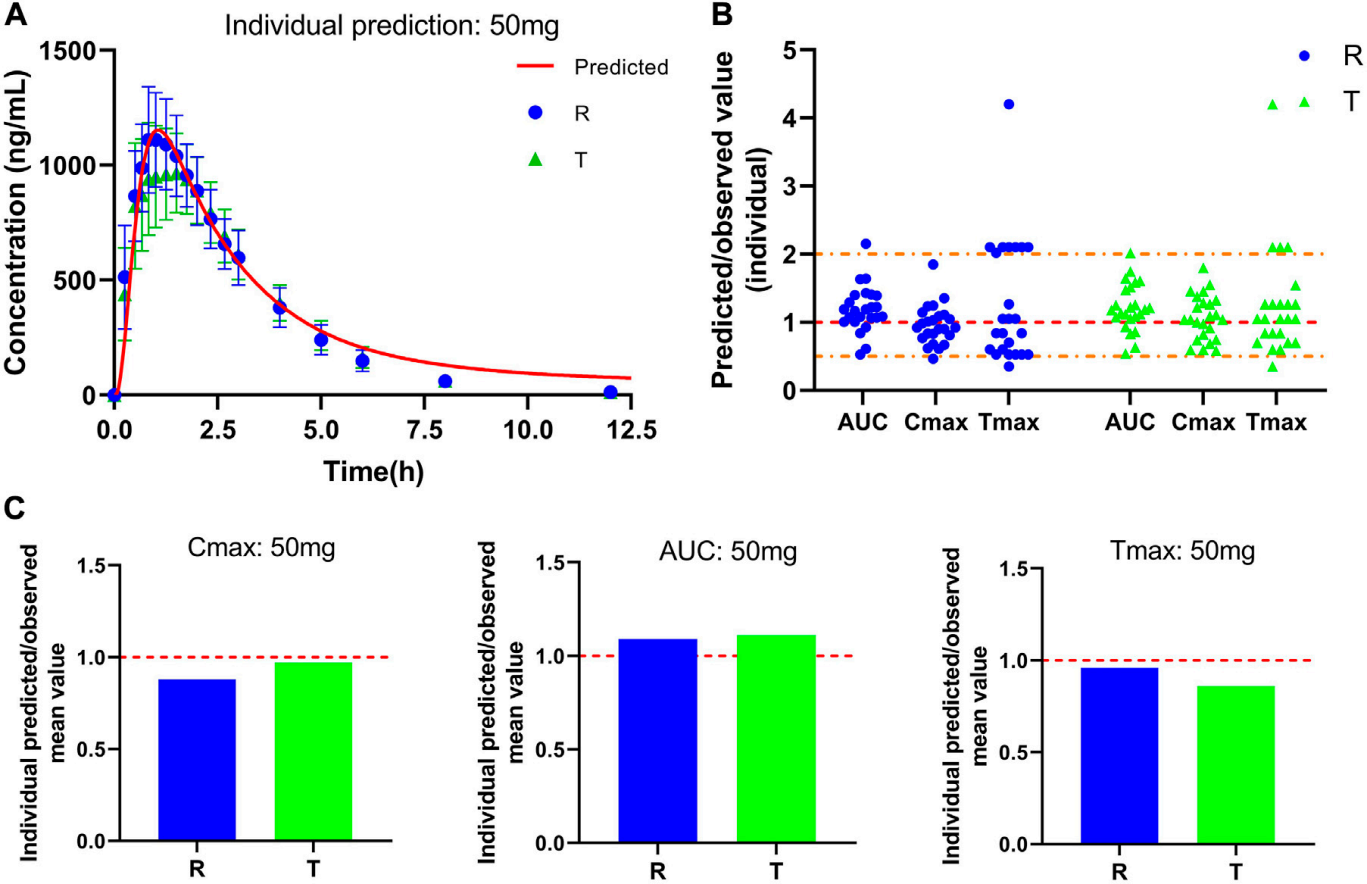
Predicted and observed plasma concentration-time curve and pharmacokinetic parameters after a single oral dose of 50 mg PTU. **(A)** Predicted plasma concentration-time curve and observed value, red solid line: predicted individual curve, blue circles: observed value (R) and standard deviation, green triangles: observed value (T) and standard deviation. **(B)** Predicted/observed value (individual), yellow dashed line: 0.5 and 2-fold error line, red dashed line: 1-fold error line. **(C)** Individual Predicted/observed mean value, red dashed line: 1-fold error line. Data are presented as mean ± standard deviation.

**FIGURE 4:**
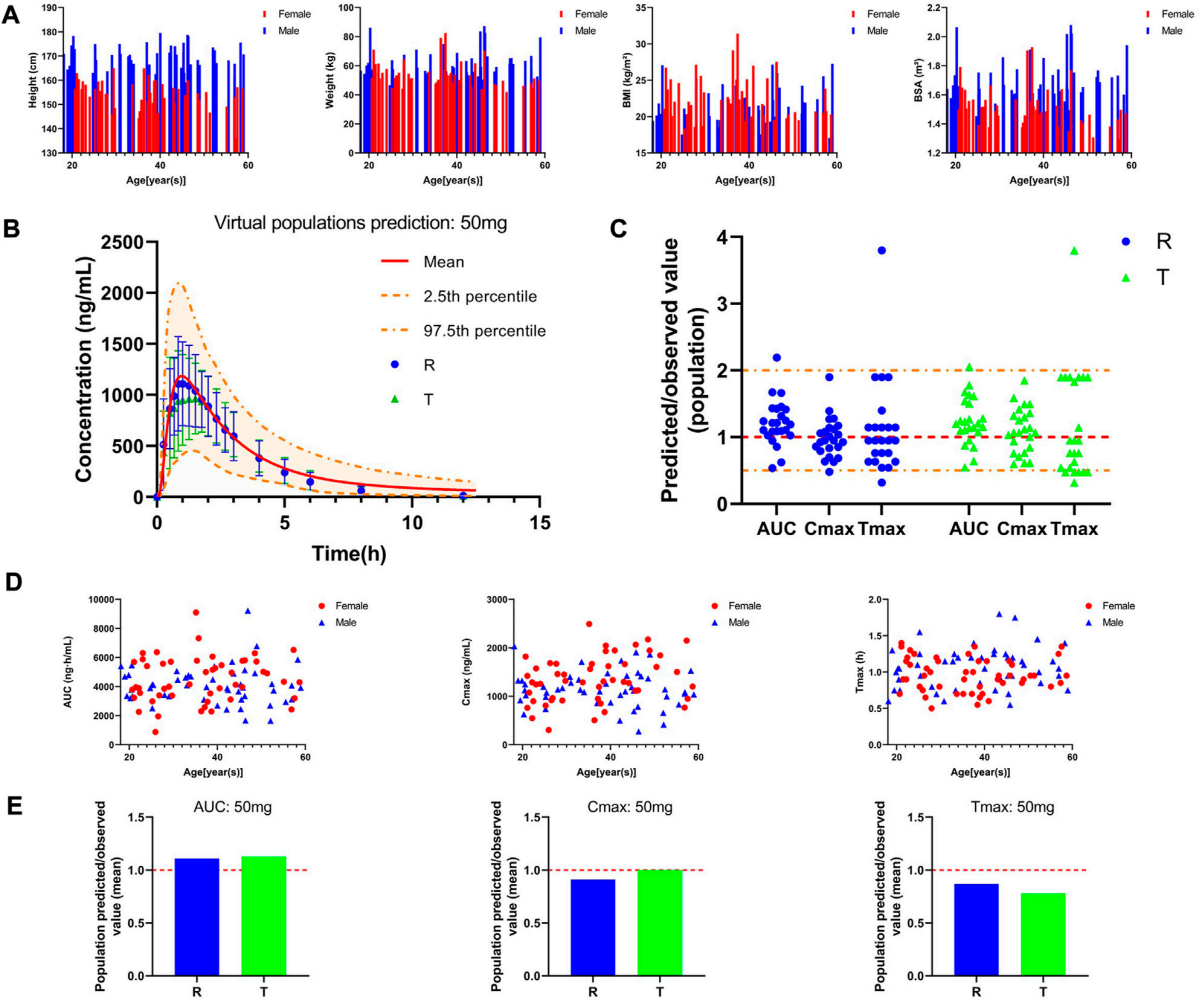
Pharmacokinetic characteristics of the virtual adult population after oral administration of 50 mg PTU. **(A)** Individual information of the virtual adult population; **(B)** Predicted plasma concentration-time curve in the virtual adult population and observed value, red solid line: predicted population mean curve, yellow dashed lines and areas: 97.fifth and 2.fifth percentile predicted values and 95% CI of predicted values, blue circles: observed value (R) and 2✕standard deviation, green triangles: observed value (T) and 2✕standard deviation. **(C)** Predicted/observed value (population), yellow dashed line: 0.5 and 2-fold error line, red dashed line: 1-fold error line. **(D)** Individual pharmacokinetic parameters in the virtual population. **(E)** Predicted/observed value (mean), red dashed line: 1-fold error line.

Plasma concentration-time curve data of healthy subjects were obtained referring to the simulated data and fitted to the actual observations to establish a typical model, and the results are shown in Figure 3A. As Figure 3B shows that comparing the predicted and observed values, a total of approximately 85% of the PK parameter values in the R cohort satisfied the condition of 0.5 < MFE< 2, and a total of approximately 92% of the PK parameter values in the T cohort satisfied the condition. In addition, compared to the typical individual predicted values and observed mean values of pharmacokinetic parameters, the MFE values are also by the requirements (Figure 3C). Meanwhile, we created a virtual population of adults containing 100 healthy subjects (50% female), aged 18–60 years, with height, weight, BMI, and BSA for each individual, as shown in Figure 4A. In Figures 4B,D, we simulated the plasma concentration-time curve data and individual pharmacokinetic parameters obtained, showing the range of 95% CI and comparing with the observed values. As shown in Figure 4C, comparing the predicted and observed mean values, approximately 94% of PK parameter values in the R cohort and approximately 89% of PK parameter values in the T cohort satisfy the condition 0.5 < MFE< 2. The MFE of the population’s mean predicted and observed values also fit the range (Figure 4E). The pharmacokinetic parameters of individual and population predicted and observed values are shown in Table 4. In summary, the virtual population PBPK model is well constructed and can be used for follow-up research.

**TABLE 4:** Predicted and observed values pharmacokinetic parameters of 50 mg PTU orally.

	Parameter	Predicted	R (MFE)	T (MFE)
Individual	AUC_0-t_ (ng*h/mL)	4199.36	3837.71 (1.09)	3770.88 (1.11)
	T_max_ (h)	1.05	1.09 (0.96)	1.22 (0.86)
	C_max_ (ng/ml)	1153.56	1308.29 (0.88)	1187.75 (0.97)
Population	AUC_0-t_ (ng*h/mL)	4278.05	3837.71 (1.11)	3770.88 (1.13)
	T_max_ (h)	0.95	1.09 (0.87)	1.22 (0.78)
	C_max_ (ng/ml)	1185.04	1308.29 (0.91)	1187.75 (1.00)

### 3.3 PBPK model validation

**FIGURE 5:**
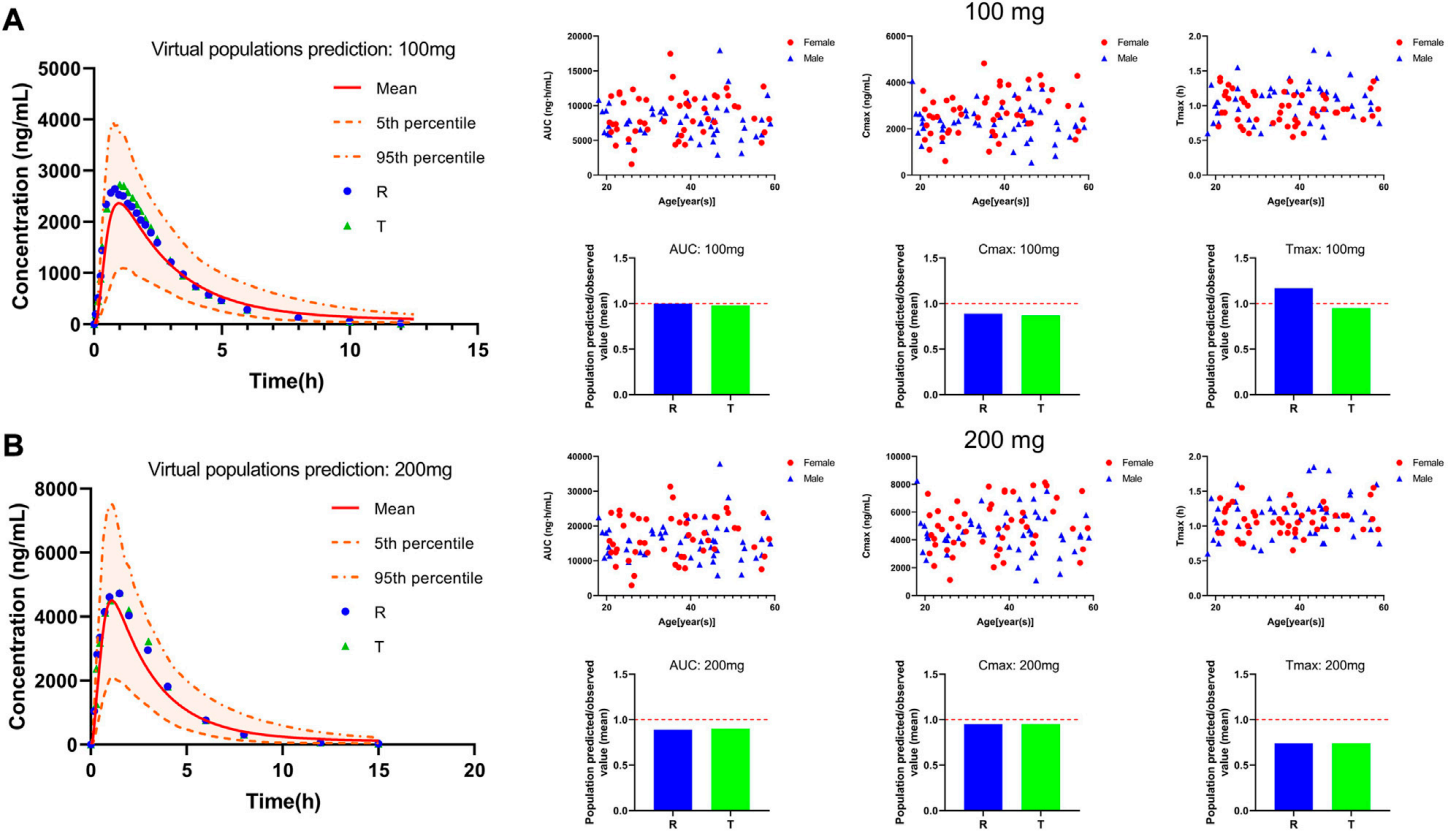
PTU 100 and 200 mg virtual adult population PBPK model validation. **(A)** 100 mg plasma concentration-time curve and pharmacokinetic parameters information. **(B)** 200 mg plasma concentration-time curve and pharmacokinetic parameters information. Red solid line: predicted population mean curve, yellow dashed lines and areas: 95th and fifth percentile predicted values and 90% CI of predicted values.

We used the mean values of two BE trials reported in the literature to validate our PBPK model to assess its reliability of the PBPK model in adults. The two studies used PTU at doses of 100 mg and 200 mg. Plasma concentration profiles were simulated in a virtual adult population at 100 mg and 200 mg of PTU orally without changing parameters other than dose. The predicted *versus* observed values of the pharmacokinetic parameter values are shown in Table 5, with MFE values in the range of 0.5 < MFE <2. The results of the PBPK model fitting for different dosing regimens are shown in Figure 5, with approximately 92% of the data within the 90% CI at the 100 mg dose and approximately 96% of the data within the 90% CI at the 200 mg dose. The above results indicate that the established PTU adult PBPK model provides a reliable basis for extrapolation studies in special populations.

**TABLE 5:** Predicted and observed pharmacokinetic parameters of 100 and 200 mg PTU orally in the virtual adult population.

Dose (mg)	Parameter	Predicted	R (MFE)	T (MFE)
100	AUC_0-t_ (ng*h/mL)	8243.16	8269.13 (1.00)	8428.67 (0.98)
	T_max_ (h)	0.95	0.81 (1.17)	1.00 (0.95)
	C_max_ (ng/ml)	2363.08	2641.98 (0.89)	2728.40 (0.87)
200	AUC_0-t_ (ng*h/mL)	16207.64	18118.41 (0.89)	17917.61 (0.90)
	T_max_ (h)	1.10	1.49 (0.74)	1.49 (0.74)
	C_max_ (ng/ml)	4510.39	4756.14 (0.95)	4728.47 (0.95)

### 3.4 PBPK model predicts the distribution of PTU in tissues

**FIGURE 6:**
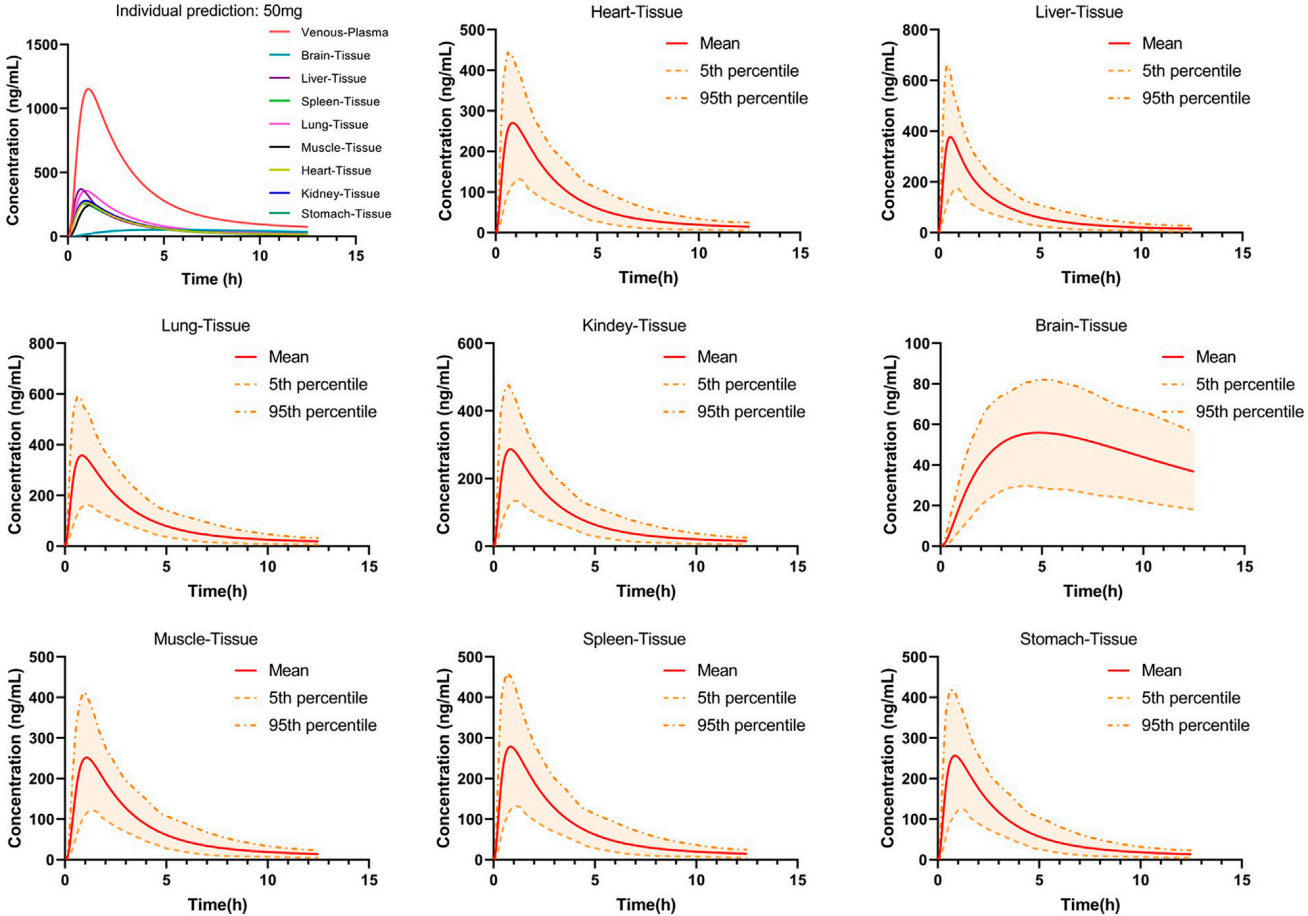
Predicted concentration-time curves of orally administered 50 mg PTU in vital tissues in a virtual adult population. Red solid line: predicted population mean curve, yellow dashed lines and areas: 95th and fifth percentile predicted values and 90% CI of predicted values.

The distribution of PTU in various tissues was predicted using the PBPK model developed and validated for plasma, as shown in Figure 6. The results showed that the tissue concentrations were lower than the plasma concentration. Comparing between major tissues, AUC_0-t_ and C_max_ were higher in liver and lung with 1,054.24 ng*h/mL, 377.37 ng/ml and 1,254.03 ng*h/mL, 357.70 ng/ml, respectively. In addition, PTU reached the peak concentration in liver tissue earlier and slightly lagged in the distribution to brain tissue with T_max_ of 0.6 h and 4.85 h, respectively. The pharmacokinetic parameters in tissues are shown in Table 6.

**TABLE 6:** Pharmacokinetic parameters of 50 mg PTU in each tissue.

Parameter	Plasma	Brain tissue	Heart tissue	Kidney tissue	Liver tissue	Lung tissue	Muscle tissue	Spleen tissue	Stomach tissue
AUC_0-t_ (ng*h/mL)	4278.15	550.88	946.21	1002.99	1054.24	1254.03	908.64	975.76	896.03
T_max_ (h)	0.95	4.85	0.85	0.85	0.60	0.85	1.05	0.85	0.85
C_max_ (ng/ml)	1185.05	55.95	270.67	286.56	377.37	357.70	251.89	278.95	256.40

### 3.5 PBPK model predicts pharmacokinetic behavioral characteristics of PTU in elderly and pediatric populations

**FIGURE 7:**
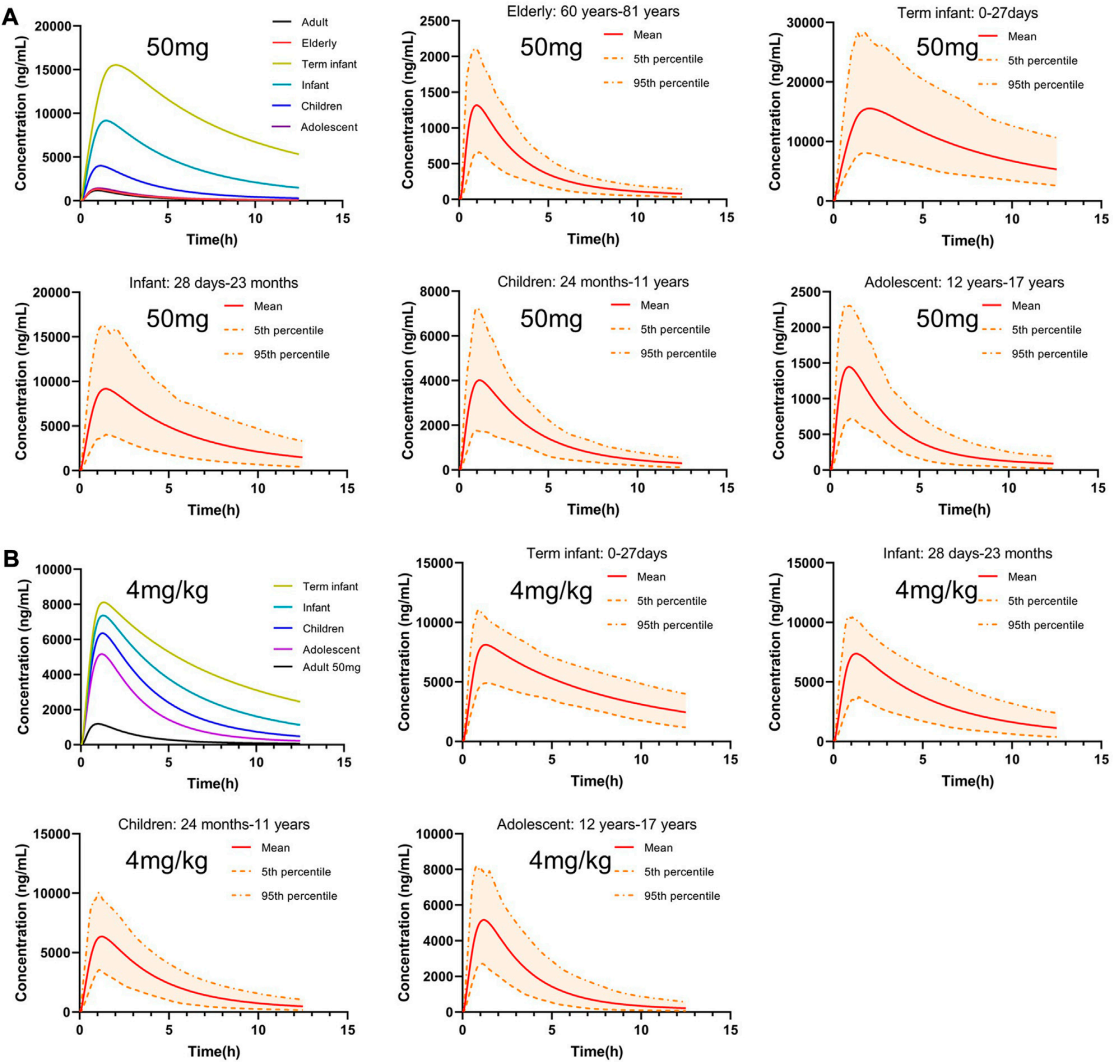
Predicted concentration-time profiles after oral administration of 50 mg or 4 mg/kg PTU in virtual healthy elderly and pediatric populations. **(A)** Predicted concentration-time profiles after oral administration of 50 mg PTU. **(B)** Predicted concentration time profiles after oral administration of 4 mg/kg PTU in virtual pediatric populations. Red solid line: predicted population mean curve, yellow dashed lines and areas: 95th and fifth percentile predicted values and 90% CI of predicted values.

Scaling clearance and fu based on the allometric scaling model and albumin concentration and obtaining *in vivo* exposure information for different populations at the same dose. The established adult oral 50 mg PTU PBPK model was extrapolated to a virtual healthy elderly and pediatric population, as shown in Figure 7A. AUC_0-t_, C_max_, and T_max_ were calculated for the elderly and pediatric populations and compared with adult parameters. As shown in Table 7, PTU exposure was significantly higher in term infants, infants, and children than that in adults, with the AUC_0-t_ being approximately 28.32, 12.65 and 4.15 folds, respectively, and the C_max_ being approximately 13.11, 7.74, and 3.39 folds, respectively than in adult population. While the exposure of the adolescent and elderly populations were essentially similar to that of adults. In addition, we also developed the PBPK models for the pediatric population at the clinically recommended starting dose of 4 mg/kg, as shown in Figure 7B. The results showed that PTU exposure in term infants, infants, children and adolescent groups were all higher than that of the adult 50 mg dose, with the AUC_0-t_ being approximately 14.99, 10.43, 7.05 and 4.74 folds, respectively, and the C_max_ being approximately 6.85, 6.22, 5.36 and 4.36 folds, respectively than in adult population. It implies that there is also some risk associated with dosing according to body weight at mg/kg, especially in the overweight children and adolescent population.

**TABLE 7:** Pharmacokinetic parameters of 50 mg or 4 mg/kg PTU orally in healthy elderly and pediatric populations.

Dose	Parameter	Adult	Elderly	Term infant	Infant	Children	Adolescent
50 mg	AUC_0-t_ (ng*h/mL)	4278.05	5021.03	121152.07	54131.18	17775.04	5658.85
	T_max_ (h)	0.95	1.00	2.00	1.45	1.15	1.05
	C_max_ (ng/ml)	1185.04	1317.97	15539.52	9171.39	4016.91	1446.26
4 mg/kg	AUC_0-t_ (ng*h/mL)	—	—	64141.05	44606.55	30155.37	20291.40
	T_max_ (h)	—	—	1.30	1.25	1.25	1.20
	C_max_ (ng/ml)	—	—	8116.81	7367.48	6350.62	5168.29

## 4 DISCUSSION

PBPK modeling and simulation approaches have gained popularity in recent years, particularly for predicting the impact of drug-drug interactions, selecting an optimal dose and clinical trial design for special populations, and simulation of tissue distribution characteristics of drugs *in vivo* (Zhao et al., 2012; Jiang et al., 2013; Lagishetty et al., 2016). In this study, the PBPK model was used to predict PTU tissue distribution characteristics and extrapolate and predict exposure in special populations of the elderly and pediatrics, offering the possibility of using the PBPK model to guide PTU dosing regimens in the future.

A whole-body PBPK model of PTU for adults has been built and evaluated by describing and predicting venous plasma concentration-time profiles and the total hepatic and renal clearance of PTU *in vivo* after oral administration. The dataset utilized a wide range of doses (50, 100, and 200 mg). After a comprehensive evaluation, the adult PBPK model was successfully developed. The PBPK model takes complete account of individual anatomical and physiological parameters and is based on material balance, which can describe the dynamic changes of drugs from arterial blood into the heart, brain, liver, kidney, spleen, and muscle tissues from each tissue into venous blood. In this study, a PTU PBPK model was developed using *in vivo*, *in vitro*, and model prediction data, by which the drug distribution in the tissue characteristics was predicted. The results showed that the concentrations in major tissues were lower than venous plasma concentrations, including liver tissue. Studies reported that a large number of drugs had been associated with liver injury and their mechanisms of hepatotoxicity are less elucidated. Characteristically, these types of drug-induced liver injury (DILI) are uncommon, unrelated to drug dose, and multifactorial dependent; therefore, these reactions are termed ‘idiosyncratic’ DILI (IDILI) (Hussaini and Farrington, 2007). Many drugs have been identified to cause IDILI in humans (Bissell et al., 2001). PTU induced hepatic injury is categorized as IDILI (Woeber, 2002). Notably, the C_max_ and AUC_0-t_ of PTU were higher in the liver compared to other tissues, which means that dose exposure levels are still a non-negligible factor.

The issue of rational drug use in special populations has always been a major clinical challenge. Due to significant individual differences, ethical and other constraints, pharmacokinetic and pharmacodynamic studies of most drugs in special populations such as the elderly and pediatrics are less reported, and there is less basis for a reference dosing regimen for special populations (Krekels et al., 2017). The product labels are generally formulated for the adult population and lack information on dosing in special populations. Such groups are likely to have potential dosing risks if administered according to the instructions. Therefore, drug regulatory authorities and academia, among others, are increasingly using the PBPK model to improve the safety of medication use in special populations.

For the pediatric population, the maturity of their organs and blood flow rates, the maturity of metabolic enzymes, the elimination pathways of drugs, and individual development are different at different ages (Shi. et al., 2018). The first drug to be recognized by FDA for clinical review and approval applying the PBPK model was retinoic acid, a highly teratogenic topical anti-wrinkle cream (Clewell et al., 1997; Rowland et al., 2011). FDA required PBPK modeling considering fetal exposure that might cause postnatal defects. In recent years, there has been an increasing number of novel and generic drugs using PBPK modeling in the drug development process. It has been reported that PTU is readily absorbed and extensively metabolized, and the pharmacokinetic behaviors of PTU *in vivo* are linear (Halpern et al., 1983). In the existing PTU dosing regimen, the starting dose for adults is generally 300 mg per day, ranging from 150 to 400 mg depending on the severity of the disease, with a maximum of 600 mg per day, gradually reduced after the disease is controlled, and the maintenance dose is 50–150 mg per day, adjusted according to the disease condition. The starting dose for pediatric patients is 4 mg/kg per day, and the maintenance dose is reduced as appropriate. This indicates that the dose must be reduced when children are taking PTU. This study fully considered individual differences between adult and pediatric populations, and hepatic and renal clearance and fu values were extrapolated and simulated. The results showed that PTU exposure in the pediatric population was increased to varying degrees compared to that of the adult population therapeutically both at the same dose and starting dose. This explains why there is much more adverse reactions in the pediatric population (Rivkees and Mattison, 2009). Our study suggests plasma concentration monitoring and reasonable dose reduction are needed in the pediatric population when using PTU, similar to what has already been reported. In addition, we found that PTU exposure under the same dose *in vivo* gradually decreased with age until it reached the same level of exposure as in the adult population. However, due to the unique nature of the pediatric population and the potential for severe adverse drug reactions, our results need to be rationally explored in the context of additional pediatric clinical trial data.

The issue of medication safety for elderly patients is also a major clinical challenge. However, considering the prevalence of combined medication use and the coexistence of multiple underlying diseases in the elderly population due to hepatic and renal insufficiency, the disposition of medications within the elderly population is difficult to predict accurately (Gnjidic et al., 2018). Therefore, this study focused on simulations in the healthy elderly population. The results showed that the exposure of PTU in the healthy elderly population was generally consistent with that of the adult population, suggesting that no dose changes are required for the use of PTU in the elderly population that does not suffer from other diseases than hyperthyroidism, assuming that this disease does not affect PTU pharmacokinetics. This result is consistent with what has been reported (Liu and Shan, 2021). In addition, the dose of medication for elderly patients should be chosen with care, taking into account the greater chance of decreased liver, kidney, and heart function in the elderly and the greater chance of comorbid diseases or other drug therapy. In particular, the dose of medication should be reduced in those with reduced renal function. If hypothyroidism is found, the treatment plan should be adjusted.

Related studies have reported that glucuronidation mediated by uridine 50-diphospho-glucuronosyltransferases (UGTs) has been proposed as a potential metabolic pathway of PTU. *In vitro* experiments showed that the UGT1A9 inhibitor (magnolol) significant inhibition of PTU metabolism when the concentrations of various subtypes of UGTs enzyme inhibitors were 10 μM and 50 μM (Li et al., 2021). Therefore, we tried to simulate the disposition process using UGT1A9 as the main metabolic enzyme. When the parameters were optimized using a Monte Carlo algorithm, the simulations yielded Michaelis constant (K_m_) values within a controlled range (reference: 15.27 ± 7.73 µM; simulated: 17.40 µM), while the maximum rate of the metabolic reaction (V_max_) differed from those reported (reference: 352.3 ± 56.38 nmol/min/mg; simulated: 171.81 nmol/min/ml). Due to limited relevant information, we could not process the data further to verify the reliability of the data. Notably, the UGT2B4 inhibitor (fluconazole) also showed significant inhibition at high concentrations of 50 μM, while other UGT inhibitors also slightly slowed down the glucuronidation response of PTU, a phenomenon that may be related to its weaker specificity of inhibition of UGT1A9. Therefore, the related literature is still controversial.

Of note, it has been reported that thyroid hormone levels may influence the disposition process of PTU (Eichelbaum, 1976). Mechanistically, thyroid hormones can bind to numerous UGT enzymes *in vivo*, and the inconsistent spatial binding capacity of different forms of thyroid hormones to UGT enzymes leads to differences in their ability to inhibit UGT enzymes (Chen et al., 2018). Therefore, high levels of thyroid hormones in hyperthyroid patients may impact the metabolism of PTU. In addition, we found different results from different investigators studying the pharmacokinetic behavior of PTU in hyperthyroid patients. Schuppan et al. and Kampmann et al. (1974) found no significant differences in the plasma half-life and clearance of PTU between patients and normal subjects. In contrast, Vesell et al. found that a significant decrease in plasma half-life occurred after oral administration of PTU in hyperthyroid patients, which may be due to accelerated hepatic microsomal drug metabolism in hyperthyroidism (Vesell et al., 1975). Another study compared the pharmacokinetic behavior of PTU in children and adolescents with Graves’ disease in hyperthyroidism and normal thyroid function. The results showed that the C_max_, absorption rate constant (Ka) and AUC of PTU were significantly higher in the hyperthyroid state, while other pharmacokinetic parameters were not significantly different from those in the normal state (Hoffman and Miceli, 1988). The differences between studies may be due to variations in sample testing methods, but it is still unclear what hyperthyroidism status’s exact effect on PTU metabolism *in vivo*.

Although the PTU PBPK model in this study has a stable structure and accurate predictions, it still has some limitations. For instance, this study only explored the oral administration of PTU and no IV data were used for model building. In addition, the extrapolation of the clearance process of PTU in this study was performed using the allometric scaling method, which was validated by external data from adults and was reasonably extrapolated but without further exploration of relevant metabolic enzymes and transporters. Moreover, while PBPK models can make reasonable extrapolations of drug concentrations in various populations and their tissues and organs, they need to be validated by external data to determine whether the model predictions are accurate. Subsequently, we will extend the results by conducting relevant basic and clinical studies to allow a better understanding and future model refinements potentially.

## 5 Conclusion

In summary, the PBPK model developed using PK-Sim® software can effectively predict *in vivo* pharmacokinetic characteristics after PTU administration and will provide a valuable reference for individualized clinical dosing and evaluation.

## Data Availability

The original contributions presented in the study are included in the article/Supplementary Material, further inquiries can be directed to the corresponding authors.
